# Hypertensive disorders of pregnancy: Case definitions & guidelines for data collection, analysis, and presentation of immunization safety data

**DOI:** 10.1016/j.vaccine.2016.03.038

**Published:** 2016-12-01

**Authors:** Caroline E. Rouse, Linda O. Eckert, Blair J. Wylie, Deirdre J. Lyell, Arundhathi Jeyabalan, Sonali Kochhar, Thomas F. McElrath, The Brighton Collaboration Preeclampsia Working Group

**Affiliations:** a Brigham and Women's Hospital, Boston, MA, USA; b University of Washington, Seattle, WA, USA; c Massachusetts General Hospital, Boston, MA, USA; d Stanford University, Stanford, CA, USA; e University of Pittsburgh, Pittsburgh, PA, USA; f Global Healthcare Consulting

**Keywords:** Preeclampsia, Hypertension in pregnancy, Adverse event, Immunization, Guidelines, Case definition

## 1 Preamble

### 1.1 Need for developing case definitions and guidelines for data collection, analysis, and presentation for the hypertensive disorders of pregnancy as adverse events following immunization

There is no universally accepted case definition of gestational hypertension, preeclampsia or eclampsia that occurs following immunisations. This is a missed opportunity, as data comparability across trials or surveillance systems would facilitate data interpretation and promote the scientific understanding of the event. As immunization is considered an essential element of care in pregnancy, the potential complications of this procedure should be understood. Additionally, vaccine studies may be conducted in a variety of settings, including those with fewer resources to perform the same diagnostic testing as in higher resource settings. It is important to provide definitions that can be utilized widely.

Around 10% of all pregnant women will be affected by a hypertensive disorder during pregnancy [1]. Hypertensive disorders of pregnancy are a significant contributor to maternal and neonatal morbidity and mortality, and are implicated in 10–15% of maternal deaths worldwide [1], [2]. The exact mechanism responsible for hypertensive diseases of pregnancy, in particular preeclampsia, is not known. One leading hypothesis is that abnormalities in the development of the uteroplacental unit lead to increased hypoxemia and oxidative stress, which in turn lead to endothelial dysfunction and abnormalities in vascular tone and coagulation [3], [4].

Hypertensive disease in pregnancy encompasses a spectrum of conditions, including gestational hypertension, preeclampsia (which can be further qualified as having severe features), eclampsia, chronic hypertension with superimposed preeclampsia and HELLP (Hemolysis, Elevated Liver Enzymes, Low Platelets) syndrome. Because of differences among the guidelines issued by international societies, diagnosis can occasionally become confusing as terminology may vary. Nevertheless, it is important to differentiate hypertensive disorders that predate pregnancy from those that occur during pregnancy, as well as to categorize patients into more or less serious cases. Furthermore, the specific diagnosis has important treatment implications, such as timing of delivery. The definitive treatment for hypertensive diseases of pregnancy is delivery.

The association of vaccination with the hypertensive diseases of pregnancy has not been well studied and the exact incidence is not known. There are observational studies as well as case reports of hypertensive disease developing in women after vaccine administration but no causal link has been described. Furthermore, the case definitions for the observational studies are not well defined, with several studies relying solely on ICD-9 codes.

### 1.2 Methods for the development of the case definition and guidelines for data collection, analysis, and presentation for the hypertensive disorders of pregnancy as adverse events following immunization

Following the process described in the overview paper [5] as well as on the Brighton Collaboration Website, http://www.brightoncollaboration.org/internet/en/index/process.html, the Brighton Collaboration *Preeclampsia Working Group* was formed in 2015 and included members with clinical, academic and public health background. The composition of the working and reference group as well as results of the web-based survey completed by the reference group with subsequent discussions in the working group can be viewed at: http://www.brightoncollaboration.org/internet/en/index/working_groups.html.

To guide the decision-making for the case definition and guidelines, a literature search of publications in English was performed using Medline, Embase and the Cochrane Libraries, including the terms vaccines, vaccination, or immunization (or terms beginning with vaccin-, immuni-, inoculat-), and [hypertension AND pregnancy] or [preeclampsia or eclampsia] (or preeclam-, eclamp-). The search resulted in the identification of 516 references. All abstracts were screened for possible reports of preeclampsia, eclampsia or hypertension in pregnancy following immunization. Twenty-seven articles with potentially relevant material were reviewed in more detail, in order to identify studies using case definitions or, in their absence, providing clinical descriptions of the case material. Data collected from these 27 articles included information on the study type, the vaccine, the diagnostic criteria or case definition put forth, the time interval since time of immunization, and any other symptoms. References that lacked hypertensive diseases of pregnancy as an outcome were excluded.

Most publications were of observational studies, though there were also several publications from vaccine adverse event reporting groups. Only one publication [6] specified the criteria used to diagnose preeclampsia in study participants. Four of the publications reported using ICD-9 diagnostic codes to collect cases of preeclampsia/eclampsia or pregnancy related hypertension [2], [7], [8], [9].

### 1.3 Rationale for selected decisions about the case definition of preeclampsia as an adverse event following immunization

#### 1.3.1 The terms for hypertension in pregnancy

The terms “eclampsia,” “preeclampsia,” “gestational hypertension” and “pregnancy-induced hypertension” are commonly used in clinical practice. “Pregnancy-induced hypertension” is a term referring to hypertensive disorders of pregnancy in general, but lacks the specificity of the other terms, and so the Brighton definitions will refer only to “eclampsia,” “preeclampsia,” and “gestational hypertension.” All of these disorders are characterized by elevations in blood pressure. Preeclampsia and eclampsia have additional diagnostic criteria based on laboratory findings by clinical physical exam or patient reported symptoms reflecting the systemic nature of the disease. The diagnosis of gestational hypertension is provisional, in that every woman with new blood pressure elevation in pregnancy should be further evaluated for the development of preeclampsia. It is possible to move from a diagnosis of gestational hypertension to preeclampsia or eclampsia, but not from preeclampsia to gestational hypertension.

#### 1.3.2 Formulating a case definition that reflects diagnostic certainty: weighing specificity versus sensitivity

The number of symptoms and/or signs that will be documented for each case may vary considerably. The case definitions have been formulated such that the Level 1 definition is highly specific for the condition. As maximum specificity normally implies a loss of sensitivity, one additional diagnostic levels have been included in the definition, offering a stepwise increase of sensitivity from Level 1 down to Level 2, while retaining an acceptable level of specificity at all levels. In this way it is hoped that all possible cases of the hypertensive diseases of pregnancy can be captured.

It needs to be emphasized that the grading of definition levels is entirely about diagnostic certainty, not clinical severity of an event. Thus, a clinically very severe event may appropriately be classified as Level 2 rather than Level 1 if it could reasonably be ascribed to an etiology other than the hypertensive diseases of pregnancy. Detailed information about the severity of the event should additionally be recorded, as specified by the data collection guidelines.

#### 1.3.3 The timing of development of preeclampsia in the context of vaccine administration

Preeclampsia and gestational hypertension are conventionally defined as developing after 20 weeks gestation [10], but there can be great variability in exact timing of presentation of the disease. In one study, approximately 10% of the preeclampsia diagnoses were made before 34 weeks gestation [11]. Preeclampsia can develop up to 6 weeks postpartum and, in fact, 20–50% of eclampsia occurs in the postpartum period [12], [13]. The progression from normal blood pressure to hypertension to preeclampsia can proceed rapidly, gradually, or not at all. Because of the unpredictability in development and progression of the disease, it is important for the purpose of vaccine trials to record the temporal relationship between immunization and development of any preeclampsia-related complication of pregnancy.

#### 1.3.4 Rationale for individual criteria related to the case definition

##### 1.3.4.1 Gestational hypertension

Gestational hypertension refers to new onset hypertension after 20 weeks of gestation [10], [14], [15]. The use of “20 weeks gestation” as a diagnostic criterion is somewhat arbitrary, as there is no specific physiologic change known that occurs at this gestational age that permits the development of preeclampsia. However, given that this convention is widely used, the Brighton Collaboration will continue to utilize it for the sake of continuity.

Accurate blood pressure measurement is fundamental for the diagnosis of a hypertensive disorder of pregnancy. The WHO released a document in 2003 detailing the proper protocols and techniques that should be utilized when measuring blood pressure. While it is outside the scope of this document to present a comprehensive guide to accurate blood pressure measurement, several important points should be highlighted. Regardless of the type of device used to measure blood pressure, accuracy should be checked regularly by comparing the measurement device to a calibrated device, and health care providers should be properly trained in taking blood pressure measurements. Blood pressure should be measured with the patient in a seated position, with the arm at the level of the heart. An appropriate cuff size should be chosen based on the patient's size (generally a length that is 1.5 times the circumference of the patient's arm). The systolic blood pressure is the pressure at which the first sounds can be heard. The disappearance of sounds, or the fifth phase, is the best measurement of diastolic blood pressure.

Blood pressure is considered elevated if the systolic blood pressure is ≥140 mmHg or the diastolic blood pressure is ≥90 mmHg, sustained over time. The length of time that the blood pressure should remain elevated varies as well, from 15 min [16] to 4 h depending on which organization guidelines are followed [10]. The Brighton Collaboration favors a longer time interval of sustained blood pressure elevations. However, with respect to the potential logistical concerns in some settings of keeping a woman for observation for several hours, we propose that a diagnosis of hypertension be made if the systolic blood pressure is ≥140 mmHg or the diastolic blood pressure is ≥90 mmHg on two measurements at a minimum of one hour apart.

##### 1.3.4.2 Preeclampsia

Preeclampsia has conventionally been defined as the development of gestational hypertension and proteinuria after 20 weeks gestation [2], [10], [14], [15], [16]. We consider preeclampsia as a systemic condition of endothelial dysfunction in which hypertension is a primary presenting sign. Other organ systems will manifest this dysfunction in fashions specific to their physiology. Historically, microvascular dysfunction in the kidney has been recognized as proteinuria.

Proteinuria can be quantified by 24 h urine collection, a spot protein:creatinine ratio, or with urinary dipstick. Proteinuria of ≥300 mg in a 24 h urine specimen (the gold standard for measurement of proteinuria), or ≥0.30 on a spot protein:creatinine ratio, or ≥1+ on a dipstick meets the criteria for preeclampsia [2], [10], [14], [15], [16]. Routine visual dipstick urinalysis has been shown to have false positive rates at “1+” of 67–83%, and false negative rates at “nil” or “trace” of 8–18% [17]. Automated urinalysis improves the sensitivity of this test to 74% [18]. The sensitivity and specificity of the protein:creatinine ratio are higher at 93% and 92%, respectively [18]. Given the potential variation in resources available to test for proteinuria, the Brighton Collaboration will permit any of these measures of proteinuria, though 24 h urine collection and protein:creatinine ratio are preferred.

Preeclampsia can be further classified as having “severe features” with development of laboratory abnormalities or symptoms. The progression to preeclampsia with severe features represents the clinical recognition of the additional involvement of maternal organ systems. Because certain clinical findings associated with severe disease increase the morbidity and mortality of preeclampsia [19], they are included in the Brighton Collaboration definition. The diagnosis of severe preeclampsia requires new onset hypertension (as described above) and one of the following criteria enumerated below. Given the multi-system nature of preeclampsia, these will be presented by system:

NOTE that preeclampsia with severe features can be diagnosed in the presence or absence of proteinuria.

• Vascular:
∘ Severely elevated blood pressures, with systolic blood pressure ≥160 mmHg and/or diastolic blood pressure ≥110 mmHg, which is confirmed after only minutes (to facilitate timely antihypertensive treatment)
• Neurologic:
∘ Development of a severe headache (which can be diffuse, frontal, temporal or occipital) that generally does not improve with over the counter pain medications (such as acetaminophen/paracetamol)
∘ Development of visual changes (including photopsia, scotomata, cortical blindness) [20]
∘ Eclampsia, or new-onset grand mal seizures in a patient with preeclampsia, without other provoking factors (such as evidence of cerebral malaria or preexisting seizure disorder). Seizures are often preceded by headaches, visual changes or altered mental status [21]
• Hematologic:
∘ New onset thrombocytopenia, with platelet count <100,000/μL
• Gastrointestinal:
∘ New onset of nausea, vomiting, epigastric pain
∘ Transaminitis (AST and ALT elevated to twice the upper limit of normal)
∘ Liver capsular hemorrhage or liver rupture
• Renal:
∘ Worsening renal function, as evidenced by serum creatinine level greater than 1.1 mg/dL or a doubling of the serum creatinine (absent other renal disease)
∘ Oliguria (urine output <500 mL/24 h)
• Respiratory:
∘ Pulmonary edema (confirmed on clinical exam or imaging)

While complications of pregnancy such as intrauterine growth restriction, placental abruption and stillbirth are utilized as diagnostic criteria for preeclampsia with severe features by some societies [15], [16], the Brighton Collaboration has chosen not to include these in our definition since these conditions frequently exist independently of the hypertensive disorders of pregnancy and may represent a separate set of pathologies. We recommend that these complications should certainly be reported as pregnancy outcomes in the context of vaccine and other drug trials. The Brighton Collaboration working groups on stillbirth, intrauterine growth restriction and vaginal bleeding in pregnancy will have publications forthcoming to help guide diagnosis of these related conditions. http://www.brightoncollaboration.org.

#### 1.3.5 Related conditions

##### 1.3.5.1 HELLP (Hemolysis, Elevated Liver Enzymes, Low Platelets) syndrome

HELLP syndrome is considered to be a subtype of severe preeclampsia. The diagnosis is based on laboratory evaluation in which all criteria (hemolysis, liver dysfunction, thrombocytopenia) are met [22], [23]. It is important to note that hypertension may be absent in up to 15% of cases of HELLP syndrome. While we recognize HELLP as part of the preeclampsia spectrum of disease, this diagnosis is not the focus of this document, and so will not be further addressed.

##### 1.3.5.2 Chronic hypertension

Chronic hypertension refers to elevation in the systolic blood pressure to ≥140 mmHg or the diastolic blood pressure to ≥90 mmHg, sustained over a length of time (as described above) that is diagnosed either prior to pregnancy or prior to 20 weeks gestation. Hypertension that occurs in early gestation is likely to predate pregnancy, hence the establishment of 20 weeks as a boundary for the diagnosis of chronic hypertension. Chronic hypertension progresses to preeclampsia in 10–50% of cases, depending on the severity of the preexisting hypertension [24]. The diagnosis of *superimposed* preeclampsia (preeclampsia superimposed on chronic hypertension) is made based on the following criteria:

• preexisting hypertension (described above) PLUS any one of the following:
∘ new onset proteinuria (as described above)
∘ worsening of preexisting proteinuria
∘ development of any of the laboratory abnormalities or clinical findings consistent with severe preeclampsia

##### 1.3.5.3 Postpartum preeclampsia

While some of the physiologic changes of pregnancy take longer to return to a pre-pregnancy state, the postpartum period, or puerperium, encompasses the six weeks following delivery [25]. The exact incidence of new-onset postpartum preeclampsia or hypertension is difficult to measure since most women do not return to their care provider until 6 weeks after the delivery, but estimates range from 0.3% to 27%[26]. The criteria for a postpartum diagnosis of the hypertensive disorders of pregnancy are the same as the antepartum criteria.

#### 1.3.6 Timing post immunization

We postulate that a definition designed to be a suitable tool for testing associations requires ascertainment of the outcome (e.g. a hypertensive disorder of pregnancy) independent from the exposure (e.g. immunisations). Therefore, to avoid selection bias, a restrictive time interval from immunization to onset of a hypertensive disorder of pregnancy should not be an integral part of such a definition. Instead, where feasible, details of this interval should be assessed and reported as described in the data collection guidelines. Care should be taken to avoid creating spurious associations between vaccine administration and hypertensive disorders, given that vaccines are generally administered during specific times during pregnancy. Case–control studies are needed to further evaluate the potential link.

Further, hypertensive disorders of pregnancy are common, affecting up to 10% of pregnant women [1], and can occur outside the controlled setting of a clinical trial or hospital. In some settings it may be impossible to obtain a clear timeline of the event, particularly in less developed or rural settings. In order to avoid selecting against such cases, the Brighton Collaboration case definition avoids setting arbitrary time frames, though the immunization should precede the hypertensive disorder.

#### 1.3.7 Differential diagnoses

Other diagnoses should be considered during the workup of hypertension in pregnancy. The differential is broad, including but not limited to conditions such as preexisting renal disease, thrombotic thrombocytopenic purpura/hemolytic uremic syndrome, acute fatty liver of pregnancy, primary liver disease, cardiomyopathy, pheochromocytoma, and thyrotoxicosis. Seizures in pregnancy can be caused by a preexisting seizure disorder, cerebral malaria, metabolic abnormalities, or cerebral anatomic abnormalities such as a space-occupying lesion. Ensuring accurate diagnosis is of great importance, as treatment can vary widely based on the etiology of the patient's symptoms.

## 2 Case definitions

**Table:** 

**PREECLAMPSIA**
**For All Levels of Diagnostic Certainty**
**Preeclampsia** is a clinical syndrome characterized by
pregnancy ≥20 weeks
AND
new onset hypertension (systolic blood pressure ≥140 mmHg and/or diastolic
blood pressure ≥90 mmHg) sustained on two measurements over a minimum of 1 h
AND
new onset proteinuria

**Level 1 of diagnostic certainty**
proteinuria diagnosed with ≥300 mg of protein on 24 h urine collection OR
≥0.3 on spot protein:creatinine ratio

**Level 2 of diagnostic certainty**
proteinuria diagnosed with ≥1+ protein on urine dipstick

**Insufficient evidence**
blood pressure cannot be measured OR
no proteinuria evaluation is available (note diagnosis of preeclampsia with severe
features does not require proteinuria, see definition below)

**PREECLAMPSIA WITH SEVERE FEATURES**
**For All Levels of Diagnostic Certainty**
**Preeclampsia with severe features** is a clinical syndrome characterized by
pregnancy ≥20 weeks
AND
new onset hypertension (systolic blood pressure ≥140 mmHg and/or diastolic
blood pressure ≥90 mmHg) sustained on two measurements over a minimum of 1 h
AND
At least one of the criteria for severe disease:

**Level 1 of diagnostic certainty**
At least one of the following:
Systolic blood pressure ≥160 mmHg and/or diastolic blood pressure ≥110
mmHg, which is confirmed after only minutes OR
Development of severe, persistent headache OR
Development of visual changes OR
Eclampsia OR
New onset thrombocytopenia (platelets <100,000/μL) OR
New onset unremitting epigastric pain OR
AST and ALT elevated to twice upper limit of normal OR
Evidence of liver capsular hematoma or liver rupture (diagnosed on clinical exam or with imaging) OR
Worsening renal function, as evidenced by serum creatinine level greater than 1.1 mg/dL or a doubling of the serum creatinine (absent other renal disease) or oliguria (<500 cc/24 h) OR
Pulmonary edema (confirmed on imaging with chest X-ray, or on clinical exam)

**Level 2 of diagnostic certainty**
new onset nausea and vomiting

**Insufficient evidence**
blood pressure cannot be measured

**GESTATIONAL HYPERTENSION**
**For All Levels of Diagnostic Certainty**
**Gestational Hypertension** is a clinical syndrome characterized by
pregnancy ≥20 weeks
AND
new onset hypertension (systolic blood pressure ≥140 mmHg and/or diastolic
blood pressure ≥90 mmHg) sustained on two measurements over a minimum of 1 h
WITHOUT
severe features (see preeclampsia with severe features category) and WITHOUT
proteinuria

**Level 1 of diagnostic certainty**
no proteinuria (as defined by 24 h urine collection < 300 mg, spot protein:creatinine ratio <0.3)

**Level 2 of diagnostic certainty**
no proteinuria (as defined by urine dipstick negative or trace)

**Insufficient evidence**
blood pressure cannot be measured OR
no proteinuria evaluation is available

### 2.1 Guidelines for data collection, analysis and presentation

As mentioned in the overview paper, the case definition is accompanied by guidelines which are structured according to the steps of conducting a clinical trial, i.e. data collection, analysis and presentation. Neither case definition nor guidelines are intended to guide or establish criteria for management of ill infants, children, or adults. Both were developed to improve data comparability.

### 2.2 Periodic review

Similar to all Brighton Collaboration case definitions and guidelines, review of the definition with its guidelines is planned on a regular basis (i.e. every three to five years) or more often if needed.

## 3 Guidelines for data collection, analysis and presentation of the hypertensive disorders of pregnancy, as presented in document

It was the consensus of the Brighton Collaboration *Hypertensive Disorders of Pregnancy Working Group* to recommend the following guidelines to enable meaningful and standardized collection, analysis, and presentation of information about these conditions. However, implementation of all guidelines might not be possible in all settings. The availability of information may vary depending upon resources, geographical region, and whether the source of information is a prospective clinical trial, a post-marketing surveillance or epidemiological study, or an individual report of hypertension in pregnancy. Also, as explained in more detail in the overview paper in this volume, these guidelines have been developed by this working group for guidance only, and are not to be considered a mandatory requirement for data collection, analysis, or presentation.

### 3.1 Data collection

These guidelines represent a desirable standard for the collection of data on availability following immunization to allow for comparability of data, and are recommended as an addition to data collected for the specific study question and setting. The guidelines are not intended to guide the primary reporting of the hypertensive disorders of pregnancy to a surveillance system or study monitor. Investigators developing a data collection tool based on these data collection guidelines also need to refer to the criteria in the case definition, which are not repeated in these guidelines. The Brighton Collaboration has developed guidelines for data collection https://brightoncollaboration.org/public/resources/standards/guidelines.html; and data collection forms https://brightoncollaboration.org/public/resources/data-collection-forms.html.

Guidelines below have been developed to address data elements for the collection of adverse event information as specified in general drug safety guidelines by the International Conference on Harmonization of Technical Requirements for Registration of Pharmaceuticals for Human Use [27], and the form for reporting of drug adverse events by the Council for International Organizations of Medical Sciences [28]. These data elements include an identifiable reporter and patient, one or more prior immunisations, and a detailed description of the adverse event, in this case, of a hypertensive disorder of pregnancy following immunization. The additional guidelines have been developed as guidance for the collection of additional information to allow for a more comprehensive understanding of development of the hypertensive disorders of pregnancy following immunization.

#### 3.1.1 Source of information/reporter

For all cases and/or all study participants, as appropriate, the following information should be recorded:

1) Date of report.
2) Name and contact information of person reporting^2^ and/or diagnosing the hypertensive disorder of pregnancy as specified by country-specific data protection law.
3) Name and contact information of the investigator responsible for the subject, as applicable.
4) Relation to the patient (e.g., immunizer [clinician, nurse], family member [indicate relationship], other).

#### 3.1.2 Vaccinee/control

##### 3.1.2.1 Demographics

For all cases and/or all study participants, as appropriate, the following information should be recorded:

5) Case/study participant identifiers (e.g. first name initial followed by last name initial) or code (or in accordance with country-specific data protection laws).
6) Date of birth, age, and sex.
7) For infants: Gestational age and birth weight.

##### 3.1.2.2 Clinical and immunization history

For all cases and/or all study participants, as appropriate, the following information should be recorded:

8) Past medical history, including hospitalisations, underlying diseases/disorders, pre-immunization signs and symptoms including identification of indicators for, or the absence of, a history of allergy to vaccines, vaccine components or medications; food allergy; allergic rhinitis; eczema; asthma.
9) Any medication history (other than treatment for the event described) prior to, during, and after immunization including prescription and non-prescription medication as well as medication or treatment with long half-life or long term effect. (e.g. immunoglobulins, blood transfusion and immunosuppressants).
10) Immunization history (i.e. previous immunisations and any adverse event following immunization (AEFI)), in particular occurrence of a hypertensive disorder in pregnancy after a previous immunization.

#### 3.1.3 Details of the immunization

For all cases and/or all study participants, as appropriate, the following information should be recorded:

11) Date and time of immunization(s).
12) Description of vaccine(s) (name of vaccine, manufacturer, lot number, dose (e.g. 0.25 mL, 0.5 mL, etc.) and number of dose if part of a series of immunisations against the same disease).
13) The anatomical sites (including left or right side) of all immunisations (e.g. vaccine A in proximal left lateral thigh, vaccine B in left deltoid).
14) Route and method of administration (e.g. intramuscular, intradermal, subcutaneous, and needle-free (including type and size), other injection devices).
15) Needle length and gauge.

#### 3.1.4 The adverse event

16) For all cases at any level of diagnostic certainty and for reported events with insufficient evidence, the criteria fulfilled to meet the case definition should be recorded.
  Specifically document:
17) Clinical description of signs and symptoms of the hypertensive disorder of pregnancy, and if there was medical confirmation of the event (i.e. patient seen by physician).
18) Date/time of onset,^3^ first observation^4^ and diagnosis,^5^ end of episode^6^ and final outcome.^7^
19) Concurrent signs, symptoms, and diseases.
20) Measurement/testing
• Values and units of routinely measured parameters (e.g. temperature, blood pressure)–in particular those indicating the severity of the event;
• Method of measurement (e.g. type of thermometer, oral or other route, duration of measurement, etc.);
• Results of laboratory examinations, surgical and/or pathological findings and diagnoses if present.
21) Treatment given for the hypertensive disorder of pregnancy, especially any antihypertensive medication, magnesium sulfate and steroid medications.
22) Outcome^7^ at last observation.
23) Objective clinical evidence supporting classification of the event as “serious”.^8^
24) Exposures other than the immunization 24 h before and after immunization (e.g. food, environmental) considered potentially relevant to the reported event.

#### 3.1.5 Miscellaneous/general

25) The duration of surveillance for the hypertensive disorders of pregnancy should be predefined based on
• Biologic characteristics of the vaccine e.g. live attenuated versus inactivated component vaccines;
• Biologic characteristics of the vaccine-targeted disease;
• Biologic characteristics of the hypertensive disorders of pregnancy including patterns identified in previous trials (e.g. early-phase trials); and
• Biologic characteristics of the vaccinee (e.g. nutrition, underlying disease like immunodepressing illness).
26) The duration of follow-up reported during the surveillance period should be predefined likewise. It should aim to continue to resolution of the event.
27) Methods of data collection should be consistent within and between study groups, if applicable.
28) Follow-up of cases should attempt to verify and complete the information collected as outlined in data collection guidelines 1–24.
29) Investigators of patients with a hypertensive disorder of pregnancy should provide guidance to reporters to optimize the quality and completeness of information provided.
30) Reports of hypertensive disorders of pregnancy should be collected throughout the study period regardless of the time elapsed between immunization and the adverse event. If this is not feasible due to the study design, the study periods during which safety data are being collected should be clearly defined.

### 3.2 Data analysis

The following guidelines represent a desirable standard for analysis of data on the hypertensive disorders of pregnancy to allow for comparability of data, and are recommended as an addition to data analyzed for the specific study question and setting.

31) Reported events should be classified in one of the following five categories including the three levels of diagnostic certainty. Events that meet the case definition should be classified according to the levels of diagnostic certainty as specified in the case definition. Events that do not meet the case definition should be classified in the additional categories for analysis.

**Event classification in 5 categories**^9^

**Event meets case definition**

1) Level 1: Criteria as specified in the Hypertensive Disorders of Pregnancy case definition
2) Level 2: Criteria as specified in the Hypertensive Disorders of Pregnancy case definition

**Event does not meet case definitionAdditional categories for analysis**

3) Reported hypertensive disorder of pregnancy with insufficient evidence to meet the case definition^10^,^11^
4) Not a case of a hypertensive disorder of pregnancy

32) The interval between immunization and reported hypertensive disorder of pregnancy could be defined as the date/time of immunization to the date/time of onset^3^ of the first symptoms and/or signs consistent with the definition. If few cases are reported, the concrete time course could be analyzed for each; for a large number of cases, data can be analyzed in the following increments:

**Subjects with a hypertensive disorder of pregnancy by interval to presentation**

**Table:** 

Interval*	Number
<1 week after immunization	
<1 week <1 month after immunization	
1 month – <3 months after immunization	
<3 months – <6 months after immunization	
Every 3 months increments thereafter through 6 weeks postpartum	
Total

33) The duration of a possible hypertensive disorder of pregnancy could be analyzed as the interval between the date/time of onset^2^ of the first symptoms and/or signs consistent with the definition and the end of episode^6^ and/or final outcome.^7^ Whatever start and ending are used, they should be used consistently within and across study groups.
34) If more than one measurement of a particular criterion is taken and recorded, the value corresponding to the greatest magnitude of the adverse experience could be used as the basis for analysis. Analysis may also include other characteristics like qualitative patterns of criteria defining the event.
35) The distribution of data (as numerator and denominator data) could be analyzed in predefined increments (e.g. measured values, times), where applicable. Increments specified above should be used. When only a small number of cases is presented, the respective values or time course can be presented individually.
36) Data on hypertensive disorders of pregnancy obtained from subjects receiving a vaccine should be compared with those obtained from an appropriately selected and documented control group(s) to assess background rates of hypersensitivity in non-exposed populations, and should be analyzed by study arm and dose where possible, e.g. in prospective clinical trials.

### 3.3 Data presentation

These guidelines represent a desirable standard for the presentation and publication of data on hypertensive disorders of pregnancy following immunization to allow for comparability of data, and are recommended as an addition to data presented for the specific study question and setting. Additionally, it is recommended to refer to existing general guidelines for the presentation and publication of randomized controlled trials, systematic reviews, and meta-analyses of observational studies in epidemiology (e.g. statements of Consolidated Standards of Reporting Trials (CONSORT), of Improving the quality of reports of meta-analyses of randomized controlled trials (QUORUM), and of Meta-analysis Of Observational Studies in Epidemiology (MOOSE), respectively) [29], [30], [31].

37) All reported events of hypertensive disorders of pregnancy should be presented according to the categories listed in guideline 31.
38) Data on possible hypertensive disorders of pregnancy should be presented in accordance with data collection guidelines 1–24 and data analysis guidelines 31–36.
39) Terms to describe hypertensive disorders of pregnancy such as “low-grade”, “moderate”, “high”, or “significant” are highly subjective, prone to wide interpretation, and should be avoided, unless clearly defined.
40) Data should be presented with numerator and denominator (n/N) (and not only in percentages), if available.

Although immunization safety surveillance systems denominator data are usually not readily available, attempts should be made to identify approximate denominators. The source of the denominator data should be reported and calculations of estimates be described (e.g. manufacturer data like total doses distributed, reporting through Ministry of Health, coverage/population based data, etc.).

41) The incidence of cases in the study population should be presented and clearly identified as such in the text.
42) If the distribution of data is skewed, median and range are usually the more appropriate statistical descriptors than a mean. However, the mean and standard deviation should also be provided.
43) Any publication of data on the hypertensive disorders of pregnancy should include a detailed description of the methods used for data collection and analysis as possible. It is essential to specify:
• The study design;
• The method, frequency and duration of monitoring for the hypertensive disorders of pregnancy;
• The trial profile, indicating participant flow during a study including drop-outs and withdrawals to indicate the size and nature of the respective groups under investigation;
• The type of surveillance (e.g. passive or active surveillance);
• The characteristics of the surveillance system (e.g. population served, mode of report solicitation);
• The search strategy in surveillance databases;
• Comparison group(s), if used for analysis;
• The instrument of data collection (e.g. standardized questionnaire, diary card, report form);
• Whether the day of immunization was considered “day one” or “day zero” in the analysis;
• Whether the date of onset^3^ and/or the date of first observation^4^ and/or the date of diagnosis^5^ was used for analysis; and
• Use of this case definition for the hypertensive disorders of pregnancy, in the abstract or methods section of a publication.^12^

## Disclaimer

The findings, opinions and assertions contained in this consensus document are those of the individual scientific professional members of the working group. They do not necessarily represent the official positions of each participant's organization (e.g., government, university, or corporation). Specifically, the findings and conclusions in this paper are those of the authors and do not necessarily represent the views of their respective institutions.

## Footnotes

2 If the reporting center is different from the vaccinating center, appropriate and timely communication of the adverse event should occur.

3 The date and/or time of onset is defined as the time post immunization, when the first sign or symptom indicative of a hypertensive disorder of pregnancy occurred. This may only be possible to determine in retrospect.

4 The date and/or time of first observation of the first sign or symptom indicative for a hypertensive disorder of pregnancy can be used if date/time of onset is not known.

5 The date of diagnosis of an episode is the day post immunization when the event met the case definition at any level.

6 The end of an episode is defined as the time the event no longer meets the case definition at the lowest level of the definition.

7 E.g. recovery to pre-immunization health status, spontaneous resolution, therapeutic intervention, persistence of the event, sequelae, death.

8 An AEFI is defined as serious by international standards if it meets one or more of the following criteria: (1) it results in death, (2) is life-threatening, (3) it requires inpatient hospitalization or results in prolongation of existing hospitalization, (4) results in persistent or significant disability/incapacity, (5) is a congenital anomaly/birth defect, (6) is a medically important event or reaction.

9 To determine the appropriate category, the user should first establish, whether a reported event meets the criteria for the lowest applicable level of diagnostic certainty, e.g. Level two. If the lowest applicable level of diagnostic certainty of the definition is met, and there is evidence that the criteria of the next higher level of diagnostic certainty are met, the event should be classified in the next category. This approach should be continued until the highest level of diagnostic certainty for a given event could be determined. Major criteria can be used to satisfy the requirement of minor criteria. If the lowest level of the case definition is not met, it should be ruled out that any of the higher levels of diagnostic certainty are met and the event should be classified in additional categories four or five.

10 If the evidence available for an event is insufficient because information is missing, such an event should be categorized as “Reported hypertensive disorder of pregnancy with insufficient evidence to meet the case definition”.

11 An event does not meet the case definition if investigation reveals a negative finding of a necessary criterion (necessary condition) for diagnosis. Such an event should be rejected and classified as “Not a case of a hypertensive disorder of pregnancy”.

12 Use of this document should preferably be referenced by referring to the respective link on the Brighton Collaboration website (http://www.brightoncollaboration.org).
